# Long-Period Fiber Grating Sensors for the Measurement of Liquid Level and Fluid-Flow Velocity

**DOI:** 10.3390/s120404578

**Published:** 2012-04-10

**Authors:** Jian-Neng Wang, Ching-Ying Luo

**Affiliations:** Department of Construction Engineering, National Yunlin University of Science and Technology, Douliou 64002, Taiwan; E-Mail: g9916703@yuntech.edu.tw

**Keywords:** long-period fiber grating (LPFG), sensor, Shewhart control chart, refractive index (RI), wavelength shift, liquid level, fluid-flow velocity

## Abstract

This paper presents the development and assessment of two types of Long Period Fiber Grating (LPFG)-based sensors including a mobile liquid level sensor and a reflective sensor for the measurement of liquid level and fluid-flow velocity. Shewhart control charts were used to assess the liquid level sensing capacity and reliability of the mobile CO_2_-laser engraved LPFG sensor. There were ten groups of different liquid level experiment and each group underwent ten repeated wavelength shift measurements. The results showed that all measurands were within the control limits; thus, this mobile sensor was reliable and exhibited at least 100-cm liquid level measurement capacity. In addition, a reflective sensor consisting of five LPFGs in series with a reflective end has been developed to evaluate the liquid level and fluid-flow velocity. These five LPFGs were fabricated by the electrical arc discharge method and the reflective end was coated with silver by Tollen's test. After each liquid level experiment was performed five times, the average values of the resonance wavelength shifts for LPFG Nos. 1–5 were in the range of 1.35–9.14 nm. The experimental findings showed that the reflective sensor could be used to automatically monitor five fixed liquid levels. This reflective sensor also exhibited at least 100-cm liquid level measurement capacity. The mechanism of the fluid-flow velocity sensor was based on analyzing the relationship among the optical power, time, and the LPFG's length. There were two types of fluid-flow velocity measurements: inflow and drainage processes. The differences between the LPFG-based fluid-flow velocities and the measured average fluid-flow velocities were found in the range of 8.7–12.6%. For the first time to our knowledge, we have demonstrated the feasibility of liquid level and fluid-flow velocity sensing with a reflective LPFG-based sensor without modifying LPFGs or coating chemical compounds.

## 1. Introduction

The primary motivation of this study is to develop and assess a light weight and low-cost long-period fiber grating (LPFG) sensor for the measurement of liquid level and fluid-flow velocity, which has the potential for use in civil engineering work such as health monitoring for pavement structures [1] and other applications such as liquid level monitoring of tanks or reservoirs for industrial sectors, debris flow monitoring and warnings for the tropical cyclone season, as well as water level and fluid-flow velocity monitoring for hydraulic applications such as pipes, channels, and dam facilities.

The development and fabrication of LPFGs and the related measurands take place in many physical parameters, such as temperature, strain, refractive index (RI), bending, in-series, and multi-parameter sensing [2]. The LPFG in conjunction with a capillary tube can be used to measure fluid viscosity [3]. LPFGs are especially suitable for measurements and applications when liquids or solutions undergo a change in RI [2]. For liquid level sensing, a liquid level sensor has been developed using LPFG technology; the measurand is the change in RI and the liquid is oil [4]. However, there is lack of information on liquid level sensing capacity and reliability. Liquid level sensors have also been investigated using fiber Bragg grating (FBG) technology [5,6]. Other optical liquid level sensing systems include optical intensity, special D-shaped silica fiber, LED sensing, and fluorescent technologies [7–10]. The LPFG is extremely sensitive to the RI of the medium surrounding the cladding surface of the sensing grating region, thus allowing it to be used as an ambient index sensor [11–14].

For the fluid-flow velocity measurement using optical techniques, an optical fiber flowmeter has been demonstrated to use a single fiber mounted transversely to the fluid flow within the pipe. The fiber is vibrated by the natural phenomenon of vortex shedding, causing phase modulation of the optical carrier within. The flow rate is determined from the vibration frequency [15]. An optical fiber photorefractometer is reported for observing water mixed with the concentration of alcohol or sugar. The fluid-flow velocity can be estimated from the cross-correlation between outputs of two photorefractometric sensors placed at distances along the path of flow [16]. Two MEMS based optical sensors for wall shear stress and velocity measurements in flow fields are reported. The experimental results obtained with the wall shear stress sensor are compared with boundary layer velocity measurements obtained with a traversing laser Doppler anemometer [17]. A scanning laser Doppler microscope to measure the velocity flow profile within a capillary has been reported and the fluid-flow velocity is established by detecting the periodic fluctuation in the scattered light as the microspheres pass through a measurement volume with a diameter of 15 μm [18]. A novel fiber-optic fluid-flow velocity sensor based on a twin-core fiber Michelson interferometer has been proposed and the sensor only is a segment of twin-core fiber acting as cylinder cantilever beam. The force exerted on the cylinder by the slow flow speeds of order mm/s of the fluid with unknown velocity bends the fiber, which corresponds to the shift of the phase of this Michelson interferometer [19]. Summary, the above optical fluid-flow velocity sensors are based on different sensing techniques, such as two photorefractometric sensors, two MEMS based optical sensors, a traversing laser Doppler anemometer, a scanning laser Doppler microscope, and a twin-core fiber Michelson interferometer to obtain the fluid-flow velocity.

In this paper, we present the development and assessment of two types of LPFG-based sensors including a mobile liquid level sensor and a reflective sensor for the measurement of liquid level and fluid-flow velocity. Quality control charts were used to assess the liquid level sensing capacity and reliability of the mobile liquid level sensor. A reflective sensor consists of five LPFGs in series with a reflective mirror (end) has been developed to evaluate the liquid level and fluid-flow velocity without modifying LPFGs or coating chemical compounds. This reflective sensor with a reflective end and the spectrum is detected in reflective mode as a result of a reflective coating at the tip of the fiber. The reflective LPFG sensor is more suitable than a conventional transmitted LPFG sensor for sensing and monitoring applications in civil engineering. For the first time to our knowledge, we have demonstrated the feasibility of measuring liquid level and fluid-flow velocity using a reflective sensor with LPFGs in series.

## 2. Principle of Refractive Index Sensing

The LPFGs with periods ranging from several hundred microns to several millimeters couple incident light guided by a fundamental mode in the core to different forward-propagating cladding modes of high diffraction order *m* in an optical fiber, which decay rapidly because of the radiation from scattering losses. The coupling of the light into the cladding region generates a set of resonant bands centered at wavelength *λ_m_* in the transmission spectrum of the fiber. The resonance wavelengths *λ_m_* of an attenuation band are solutions of the following phase matched conditions [20]:

(1) $$\lambda_{m}=\left[n_{\text{co}}^{\text{eff}}-n_{cl,m}^{\text{eff}}\right]\Lambda =\delta n_{\text{eff}}\Lambda$$

where Λ is the period of grating, and ${\text{n}}_{\text{co}}^{\text{eff}}$ is the effective RI of the fundamental core mode at the wavelength of *λ_m_*, which also depends on the core RI and cladding RI. Besides, $n_{cl,m}^{\text{eff}}$ is the effective RI of the *m*th radial cladding mode (*m* = 2, 3, 4,….) at the wavelength *λ_m_*, which is also a function of cladding RI and in particular the RI of the surrounding medium *n_S_*. It is noted that both indices depend on the temperature and the strain experienced by the fiber. The spectral properties of individual cladding modes are determined by the fiber structure and may be observed through their associated attenuation bands. When the RI of the surrounding medium changes $n_{cl,m}^{\text{eff}}$ also changes and a wavelength shift can be obtained in the transmission spectrum. An LPFG can be very sensitive to the changes in temperature and deformations due to fiber imperfections, loading, and bending, which also produce a noticeable wavelength shift in loss peaks. Therefore, in order to precisely measure variations in RI changes, temperature changes and deformations must be compensated or avoided.

Since the grating period is unaltered under the effect of a change in ambient RI and assuming the RI of the core mode remains unchanged by *n_s_*, the influence of the variation in the RI around the cladding of an LPFG is expressed by:

(2) $${\left(\frac{d\lambda }{dn_{S}}\right)}_{m}=\left(\frac{d\lambda }{dn_{cl,m}^{\text{eff}}}\right)\left(\frac{dn_{cl,m}^{\text{eff}}}{dn_{S}}\right)$$

The spectral sensitivity, defined as *dλ/dn_s_*, is relevant to each of the measurands and contributes to the effective wavelength change of the *m*th cladding mode. The RI sensitivity of an LPFG arises from the dependence of the resonance wavelength on the effective RI of the cladding mode, which also depends on the RI of the surrounding materials. The LPFG is expected to strongly depend on the order of the coupled cladding mode because each cladding mode $\frac{dn_{cl,m}^{\text{eff}}}{dn_{s}}$ is distinct. In general, as *n_s_* increases, the spectral sensitivity increases monotonically to a maximum value occurring at the value of *n_s_* at which the cladding mode becomes unguided. The wavelength shift arising from the RI changes, for a given fiber and cladding mode, may be positive or negative, depending on the local slope of the characteristic phase-matching curve *dλ/dΛ*.

The shift is shown to be negative for all band and increases with the order of the cladding mode for this grating [12]. It is also shown that the RI of the LPFG sensitivity is chiefly within the range of 1.33 to 1.46. The distinct resonance bands disappear at approximately *n_s_* = 1.46 when the cladding mode is converted to radiation mode losses. The maximum RI sensitivity is imposed by the RI of the cladding material. No measurable wavelength shift is observed for RI above this limit. The high sensitivity of the LPFG to the surrounding medium offers the potential to monitor the chemical changes and corrosion condition for various material structures. This study is aimed to demonstrate that the wavelength shift and optical power can be implemented as a key part for LPFG-based liquid level and fluid-flow velocity sensing, respectively.

## 3. Experiment

The experiments mentioned in this section address the operation of the mobile liquid level sensor, the application of Shewhart control charts, and the description of the reflective sensor consisting of five LPFGs in series with a mirror (end) coated using Tollen's reagent for the measurement of liquid level and fluid-flow velocity. For the use of light sources, a broadband amplified spontaneous emission (ASE) light source (C + L bands, operating wavelength: 1,530 nm–1,610 nm) was used for both the mobile and reflective liquid level sensors, whereas a broadband laser diode (LD, Advantest Q81212) light source at a wavelength of 1,550 ± 30 nm was only used for the fluid-flow velocity sensor.

### 3.1. Mobile Liquid Level Sensor and Shewhart Control Chart

**Figure 1.**
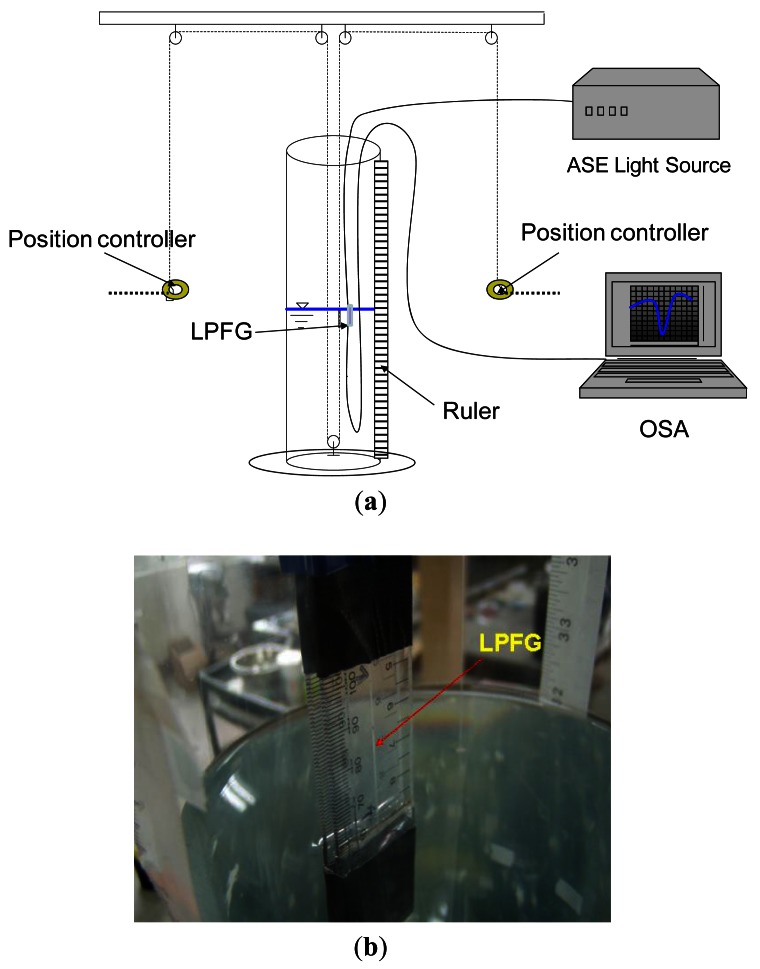
(**a**) Experimental setup for a mobile LPFG-based liquid level sensor with a 1,546.25-nm resonance wavelength LPFG and the whole LPFG was completely immersed in water for each liquid level sensing; (**b**) The photo of the mobile liquid level sensor.

The fabrication and techniques of LPFGs in this study have been reported elsewhere [11–14]. The LPFGs studied in the paper were fabricated using hydrogen-free Corning SMF-28 fibers. The CO_2_-laser engraved LPFG used for the mobile sensor was about 22 mm long and the grating period was about 550 μm. The experimental setup for the mobile liquid level sensor with a 1,546.25-nm resonance wavelength LPFG is displayed in Figure 1(a) and the top of this LPFG was exactly immersed in water for each liquid level sensing. The photo of this sensor is shown in Figure 1(b). There was a 15 cm-inside diameter, 120 cm-high, and hollow cylindrical storage tank having at least a 100-cm liquid level capacity.

**Figure 2.**
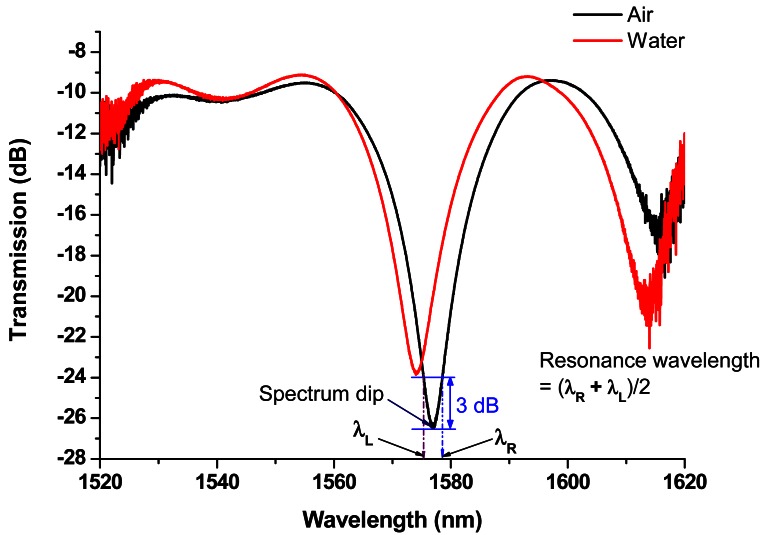
The typical transmission spectra of a CO_2_-laser engraved LPFG sensor in air and immersed in water and the resonance wavelength (1,577.06 nm) was obtained with 3-dB bandwidth method.

The position controller was established to adjust the movement of the mobile liquid level sensor. The 1,200 ± 1 mm ruler was glued on the surface of the liquid storage tank for observing different water levels. A broadband ASE light source (C + L bands: 1,530 nm–1,610 nm) and an optical spectrum analyzer (OSA, ANDO AQ6331) were used to conduct the mobile liquid level sensing measurements based on wavelength-shift detection. The measurements included the transmission spectra for different water levels. Regarding the resonance wavelength for each transmission spectrum, the 3-dB bandwidth method was used. The 3-dB bandwidth was determined by finding the dip (valley) of the spectrum, and rising by 3 dB on each side. The spectral width of the spectrum was determined by the separation of these two points because each has a power spectral density equal to one half the dip power spectral densities. The resonance wavelength was the average of two wavelengths determined in the 3-dB bandwidth measurements (see Figure 2). For precise liquid level measurements, we kept experimental setup at a constant temperature (within 0.5 °C fluctuation). Since we measured the same test temperature for both in air and water, the temperature effect causing wavelength change between room temperature and test temperature is the same and can be eliminated. The LPFG was fastened, with water-resistance adhesive tape, at both ends on a straight plastic sheet, to minimize the strain and bending effects. We controlled to minimize the variations of experimental results, not influenced by temperature, strain or bending effects, as much as possible.

**Figure 3.**
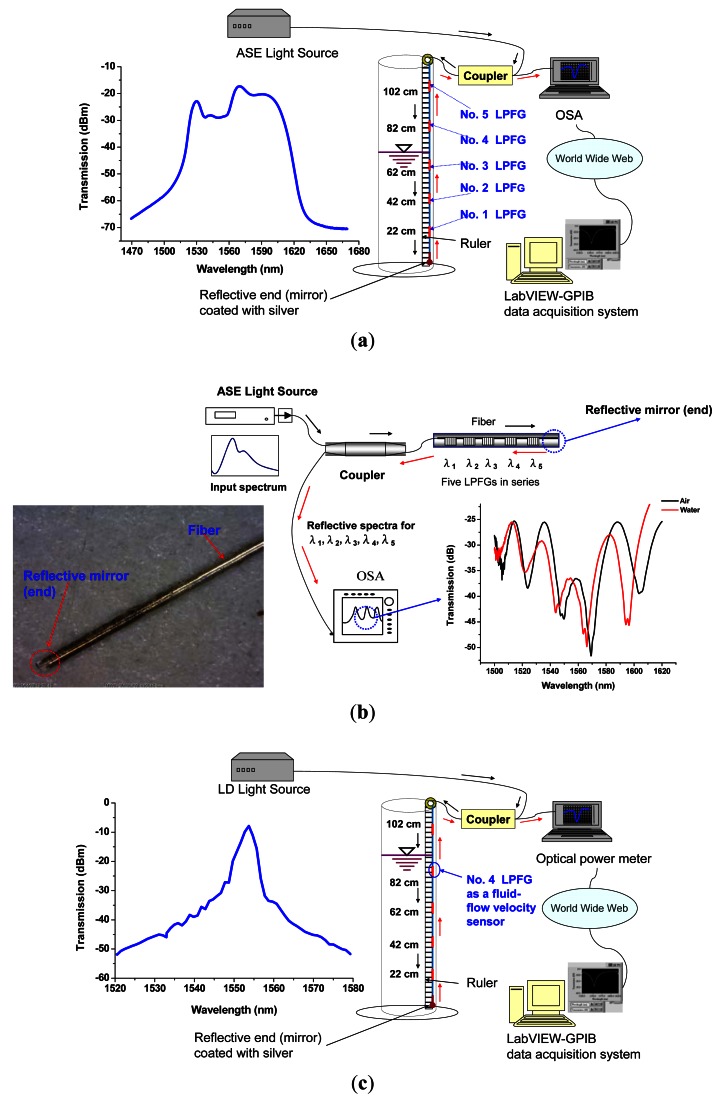
(**a**) Experimental setup for the reflective LPFGs-in-series liquid level sensor; (**b**) Schematic and photo about the reflective mode for five LPFGs in series as a result of a reflective coating at the tip of the SMF-28 fiber; (**c**) Experimental setup for the reflective fluid-flow velocity sensor.

The liquid level sensing experiment included ten groups of water levels, *i.e.*, 10, 20, 30, 40, 50, 60, 70, 80, 90, and 100 cm. For each group, the top of the LPFG was exactly immersed in water for each liquid level sensing (wavelength shift) measurements conducted 10 times for both measurements in air and immersed in water, respectively. Although the fiber was kept straight and temperature properly controlled, the 3-dB bandwidth method for spectrum dip was used to obtain the resonance wavelengths, the LPFG is also possibly sensitive to system uncertainty, such as fluctuations from the intensity of the ASE light source (see Figure 3(a): left-hand side). Thus, the results were then analyzed using the concept of Shewhart control charts, such as *X*-bar charts, *s* charts, and *R* charts [21,22], to evaluate the reliability of the mobile liquid level sensor. For new fabricated or designed products, Shewhart control charts could be used to reduce the variation, satisfy the design criteria, and improve the quality. They are often called quality control charts and could be carried out the inspection of quality improvement. A sample size of *n* of measurements is taken, and they are measured resulting in observations, *x_1_, x_2_*,…, and *x_n_*. The average *X̄* and the standard deviation *s* are computed. This is done *k* times, and the *k* values of *X̄* and *s* are averaged resulting in *X̿* and *S̄*, respectively; usually *k* is equal to some number between 10 and 30. For liquid level measurements, *k* is equal to 10.

For the average of wavelength shifts *versus* liquid level, the upper control limit (UCL) and the lower control limit (LCL) for the *X*-bar chart are expressed by:

(3) $${\text{UCL}}_{x}=\bar{\bar{X}}+A_{2}\bar{s}$$

(4) $${\text{LCL}}_{x}=\bar{\bar{X}}-A_{3}\bar{s}$$

For the *s* chart, the UCL and LCL are expressed by:

(5) $${\text{UCL}}_{s}=B_{4}\bar{s}$$

(6) $${\text{LCL}}_{x}=B_{3}\bar{s}$$

For the *R* chart, the UCL and LCL are expressed by:

(7) $${\text{UCL}}_{R}=D_{4}\bar{R}$$

(8) $${\text{LCL}}_{R}=D_{3}\bar{R}$$

where the sample size, *n* = 10, and *X*-bar, *X̄*, is the sample average and *X*-double bar, *X̿*, is the average of ten individual *X*-bar values. The *s* value is the standard deviation of the sample and s-bar, *S̄*, is the average of ten individual *s* values. *R* value is the absolute value of the difference in the extremes of the samples and *R*-bar, *R̄* is the average of ten individual *R* values. In addition, *A_2_, A_3_, B_4_, B_3_, D_4_*, and *D_3_* are the control chart constants, based on the central limit theorem and sample distribution theory, shown in Table 1 [21,22]. They can be used to determine the control limits, UCL and LCL, for the *X*-bar chart, *s* chart, and *R* chart, respectively.

### 3.2. Reflective Liquid Level Sensor

Our experimental setup for the reflective liquid level sensor is shown in Figure 3(a). The experimental devices include a liquid level sensor consisting of five LPFGs in series with a reflective end (LPFGs and the reflective end fixed on a plastic strip), a broadband ASE light source (C + L bands: 1,530 nm–1,610 nm, see Figure 3(a): left-hand side), and a computer with LabVIEW–GPIB data acquisition system connected with a high-resolution OSA (ANDO AQ6331). LPFGs were fabricated by the electrical arc discharge method [23] with hydrogen-free Corning SMF-28 fibers. The electric-arc discharge-induced LPFGs were about 1.5–3.5 cm long and their grating periods were about 600 μm. This reflective sensor with a reflective end and the spectrum was detected in reflective mode as a result of a reflective coating at the tip of the fiber. The proposed reflective liquid level sensor consisting of five LPFGs in series, resonance wavelengths: λ_1_ = 1,505.45 nm, λ_2_ =1,524.20 nm, λ_3_ =1,548.80 nm, λ_4_ =1,569.60 nm, and λ_5_ =1,605.70 nm, was fusion spliced to a reflective end (silver mirror, see Figure 3(b): left-hand side) coated with silver using Tollen's reagent [24]. When a chemical reaction exhibits an aldehyde is oxidized by silver to generate a carboxylic acid and silver metal, which could be used to coat the surface of the SMF-28 fiber. For the fabrication of silver mirror, we cleaned the test tube to be used by rinsing with concentrated nitric acid and washing well with deionized water and prepared materials as follows: 5 mL of 0.6 M AgNO_3_ (aq), 3 mL of 2.5 M NaOH (aq), 5 mL of 2 M ammonium hydroxide (aq), 10% glucose solution (C_6_H_12_O_6_). We added AgNO_3_ (aq) and NaOH (aq) into the test tube and completely mixed them together and then titrated 2 M ammonium hydroxide (aq) with stirring. We then shook this solution until the precipitated solid just dissolved completely. Later, the SMF-28 fiber was immersed inside the test tube. We added 10 drops of 10% glucose solution and the silver film was gradually formed within several minutes. A uniform coating formed on the surrounding surface of the SMF-28 fiber and a superior silver film formed on the end of the SMF-28 fiber (see Figure 3(b): left-hand side). The five LPFGs at the corresponding resonance wavelengths of λ_1_, λ_2_, λ_3_, λ_4_ and λ_5_ were interrogated using a broadband ASE light source and an OSA. A fiber coupler was used for coupling the reflected light signals of the sensor to the OSA (see Figure 3(a)). Thus, the reflective spectra of different liquid level measurements could be obtained. Figure 3(b) shows the schematic and the photo about the reflective mode for five LPFGs in series as a result of a reflective coating at the tip of the SMF-28 fiber and the marked red arrows represent the direction of reflective light. A broadband ASE light source (see Figure 3(a): left-hand side) with the home-made silver mirror (see Figure 3(b): left-hand side) were used to perform the reflective mode liquid level sensing for five LPFGs in series since we could obtain the reflective spectra (see Figure 3(a): right-hand side). Other measured reflective spectra could also be seen in the following sections; thus, our silver mirror exhibited enough reflective light intensity for the OSA to acquire the spectral data.

A 15 cm-inside diameter, 120 cm-high, and hollow cylindrical storage tank having at least a 100-cm water level capacity was used for liquid level measurements. The reflective liquid level sensing experiment included five liquid level measurements that LPFG Nos. 1–5 in series and their corresponding liquid levels were about 22, 42, 62, 82, and 102 cm marked from the bottom of the hollow cylindrical tank (see Figure 3(a)). For each liquid level, the sensing measurements were conducted five times for both in air and immersed in water, respectively. For precise liquid level and fluid-flow velocity measurements, we kept experimental setup at a constant temperature (within 0.5 °C fluctuation). Since we measured the same test temperature for both in air and water, the temperature effect causing wavelength change between room temperature and test temperature is the same and can be eliminated. The LPFGs were fastened, with water-resistance adhesive tape, at both ends on a long straight plastic sheet, which was glued to the wall of this liquid storage tank to minimize the bending of the fiber. Therefore, we controlled to minimize the variations of experimental results, not influenced by temperature, strain or bending effects, as much as possible.

### 3.3. Fluid-Flow Velocity Sensor

We used the same reflective sensor to measure the fluid-flow velocity. The mechanism of the fluid-flow velocity sensor was based on analyzing the relationship among the optical power (mW), time (s), and the LPFG's length (cm). An optical power meter was used instead of the OSA; thus, the plot of optical power *versus* time was analyzed to obtain the fluid-flow velocity (cm/s). The sensing system consists of the reflective sensor, an optical power meter (Advantest Q8221) with a broadband LD light source (Advantest Q81212) at a wavelength of 1,550 ± 30 nm (see Figure 3(c): left-hand side), and a computer with LabVIEW–GPIB data acquisition system (see Figure 3(c)). For the convenience of sight observation, we used LPFG No. 4 (around 82-cm liquid level) as a fluid-flow velocity sensor to conduct the experiment (see Figure 3(c)). There are two types of fluid-flow velocity measurements: inflow I and II (dry-to-wet process) as well as drainage (wet-to-dry process). We infused water flowing from 77-cm liquid level upward to 87-cm and 87.8-cm liquid levels for inflow I and II, respectively. The inflow experiment lasted 1,400 s and the optical power (mW) per second was acquired. The drainage flow velocity experiment was performed by discharging water flowing from 87.8-cm liquid level downward to 75.9-cm liquid level. The drainage experiment lasted 840 s and the optical power per second was also obtained. The LPFG-based fluid-flow velocity was proposed as the follows:

(9) $$v_{\text{LPFG}}=\frac{L}{\Delta_{t}}$$

where *v_LPFG_*, is the fluid-flow velocity obtained using the reflective LPFG sensor and *L* is the length of LPFG No. 4 (*L* = 1.4 cm). For the plot of optical power *versus* time, the time t_2_ and t_1_ could be obtained based on the initial and final tangential lines of a concave curve crossing the abscissa and Δ*t* is equal to t_1_ minus t_2_.

During the course of data taking, we kept the eye's horizon line be matching with the ruler and the inflow or discharge time, independently measured by using a stopwatch (within 0.1 s error), was the time duration. The measured average fluid-flow velocities, *v_Measured_*, was calculated as the ratio of fluid-flow distance (the liquid level difference) and time duration.

## 4. Results and Discussion

### 4.1. Mobile Liquid Level Measurement

**Figure 4.**
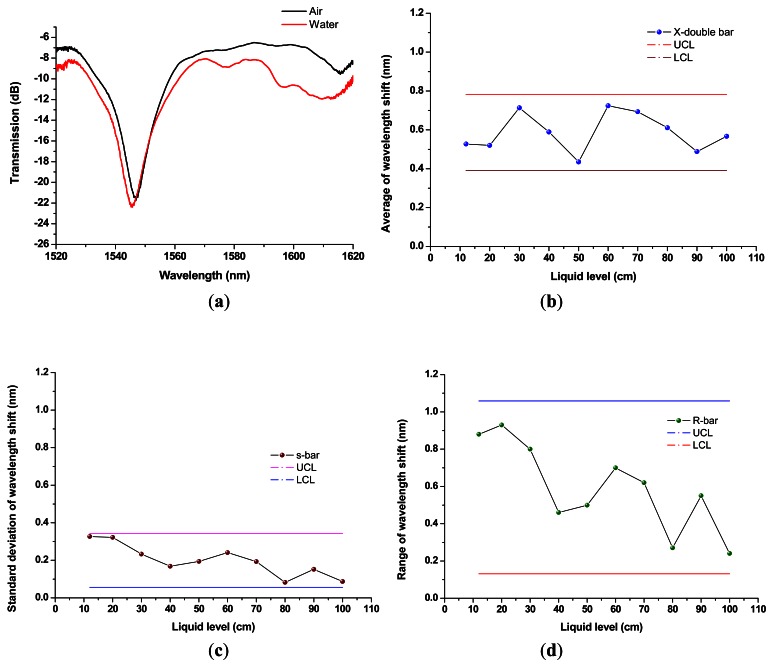
(**a**) The spectra of the mobile liquid level sensor with a 1,546.25-nm resonance wavelength LPFG was immersed in air and in water when liquid level was equal to 10 cm; (**b**) The X-bar chart of wavelength shifts for ten groups of water level experiment; (**c**) The *s* chart of wavelength shifts for ten groups of water level experiment; (**d**) The *R* chart of wavelength shifts for ten groups of water level experiment.

As described, the 100-cm liquid level capacity experiments were conducted for ten water level groups, *i.e.*, 10, 20, 30, 40, 50, 60, 70, 80, 90, and 100 cm, and each group underwent ten repeated measurements. For assessing measurement reliability, the results were analyzed and plotted using the *X*-bar chart, *s* chart, and *R* chart. The spectra of the mobile liquid level sensor with a 1,546.25-nm resonance wavelength LPFG is shown in Figure 4(a) and the sensor was immersed in air and in water when liquid level was equal to 10 cm. Figure 4(b) shows the *X*-bar chart of wavelength shifts for ten groups of water level experiment. The *s* chart and *R* chart of wavelength shifts for ten groups of water level experiment are shown in Figure 4(c,d), respectively. Each group represented 10 repeated measurements and the solid and dashed lines were UCL and LCL lines. Clearly, all measurands were within the control limits (UCL and LCL) for the *X*-bar chart, *s* chart, and *R* chart. Based on the results of the liquid level experiment using quality control charts, the mobile liquid level sensor was reliable and had 100-cm liquid level measurement capacity.

### 4.2. Reflective Liquid Level Measurement

**Figure 5.**
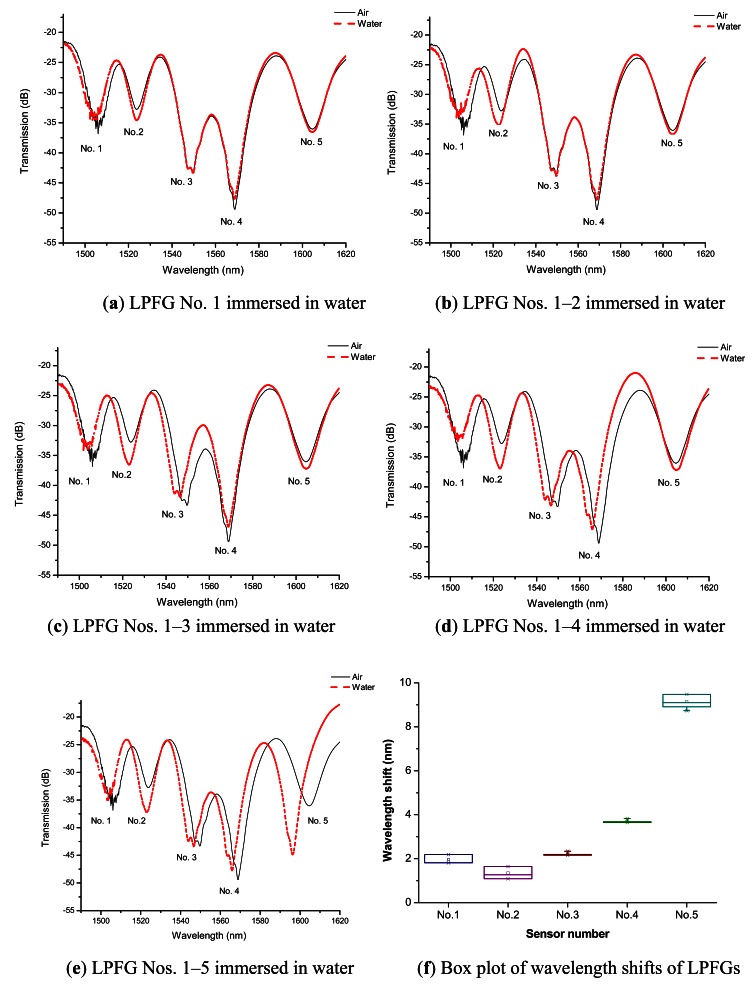
(**a–e**) Transmission spectra of the liquid level sensor with LPFG Nos. 1, 2, 3, 4, and 5 immersed in water (22, 42, 62, 82, and 102 cm liquid levels), respectively; (**f**) Box plot of average wavelength shifts by performing five times reflective liquid level measurements.

As addressed, the reflective liquid level experiment was conducted five times for both measurements in air and immersed in water, respectively. Figure 5(a–e) shows the transmission spectra of the reflective liquid level sensor with LPFG No. 1, Nos. 1–2, Nos. 1–3, Nos. 1–4, and Nos. 1–5 immersed in water, respectively. The findings showed that the reflective liquid level sensor was successfully to measure five fixed liquid levels (*i.e.*, 22, 42, 62, 82, and 102 cm). After each liquid level experiment was performed five times, the average values (with standard deviations) of the resonance wavelength shifts for LPFG Nos. 1–5 were 1.96 nm (within 0.17 nm), 1.35 nm (within 0.29 nm), 2.19 nm (within 0.07 nm), 3.68 nm (within 0.08 nm), and 9.14 nm (within 0.30 nm), respectively (see Figure 5(f)). The experimental findings show that the reflective liquid level sensor could be used to automatically monitor five fixed liquid levels and had at least 100-cm liquid level measurement capacity.

### 4.3. Fluid Flow-Velocity Measurement

**Figure 6.**
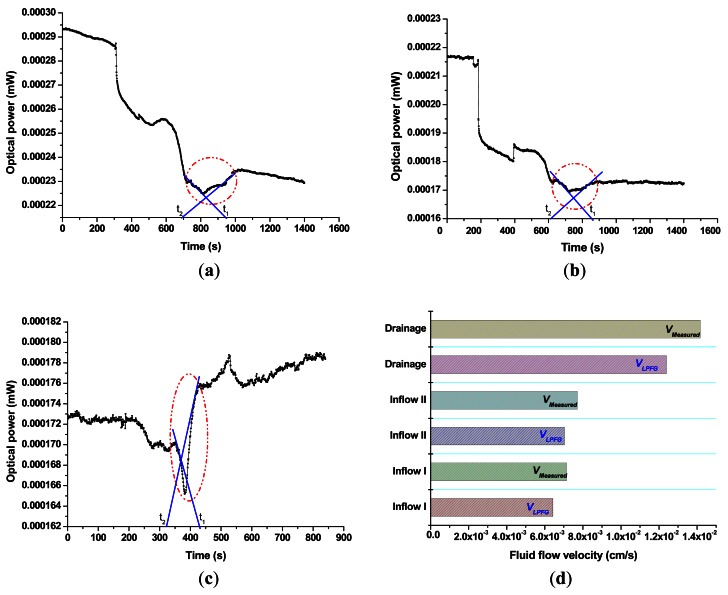
Plot of optical power *versus* time using LPFG No. 4 for fluid-flow velocity measurement: (**a**) Inflow I; (**b**) Inflow II; and (**c**) Drainage; (**d**) Comparison Plot of fluid-flow velocity for inflow and drainage processes.

Figure 6(a–c) shows the plot of optical power *versus* time for inflow I, inflow II, and drainage processes, respectively. Time t_2_ and t_1_ were obtained based on the initial and final tangential lines (blue solid lines) of a concave curve (around red dash lines) crossing the abscissa. The LPFG-based flow velocities, *v_LPFG_*, were calculated for inflow I, inflow II, and discharge processes as 6.424 × 10^−3^ cm/s, 7.043 × 10^−3^ cm/s, and 1.239 × 10^−2^ cm/s, respectively. In addition, the measured average flow velocities, *v_Measured_*, determined as the ratio of flow distance and time duration for inflow I, inflow II, and discharge processes were 7.143 × 10^−3^ cm/s, 7.714 × 10^−3^ cm/s, and 1.417 × 10^−2^ cm/s, respectively. The comparison plot of the fluid-flow velocity experiment was shown in Figure 6(d) and the differences between *v_Measured_* and *v_LPFG_* for inflow I, inflow II, and drainage processes were 10.1%, 8.7%, and 12.6%, respectively. The differences (8.7% and 10.1%, respectively) between *v_Measured_* and *v_LPFG_* for dry-to-wet cases (inflow I and II) were less than that (12.6%) of wet-to-dry process (drainage).

Based on the results, we found that the LPFG-based fluid-flow velocities, *v_LPFG_*, were less than those obtained from the measured average fluid-flow velocities, *v_Measured_* and the differences between *v_LPFG_* and *v_Measured_* were found in the range of 8.7–12.6%. In addition, the dry-to-wet process had smaller differences than those obtained from wet-to-dry condition since the LPFG sensing is more representative when the surrounding RI changes significantly. It is obvious that the reflective sensor has potential to measure liquid level and fluid-flow velocity. In addition, we demonstrated the feasibility of the reflective flow-velocity sensor and have not yet taken the error analysis and measurement uncertainty into account. Te principle of calibrating, characterizing, and accumulating uncertainties for the optical power with flow velocity usually is based on the *IEC 61315 standard—Calibration of fiber-optic power meters* [25]. This standard method takes into account the random and systematic uncertainties of all power meters and all transfer processes in the calibration chain according to *Guide to the expression of uncertainty in measurement* (GUM:1995) [26].

## 5. Conclusions

This paper presents the development and assessment of two types of LPFG-based sensors including a mobile liquid level sensor and a reflective sensor for the measurement of liquid level and fluid-flow velocity. A broadband ASE light source (1,530 nm–610 nm) was used for both the mobile and reflective liquid level sensors, whereas a broadband LD light source at a wavelength of 1,550 ± 30 nm was only used for the fluid-flow velocity sensor. Shewhart control charts, X-bar charts, s charts, and R charts, were used to assess the liquid level sensing capacity and reliability of the mobile liquid level sensor. The LPFG used for the mobile liquid level sensor was fabricated with the CO_2_-laser engraving method and this liquid level sensor was designed with the capacity of moving upward and downward using a position controller. There were ten groups of different liquid level capacity experiment and each group underwent ten repeated wavelength shift measurements. The results of Shewhart control charts showed that all measurands were within the upper and lower control limits. The examined mobile liquid level sensor was reliable and had at least 100-cm liquid level measurement capacity.

A reflective liquid level sensor consisting of five LPFGs in series with a reflective end (silver mirror) has been demonstrated to evaluate the liquid level and fluid-flow velocity. The LPFGs used for reflective liquid level and fluid-flow velocity sensors were fabricated by the electrical arc discharge method. This reflective sensor was fusion spliced to a reflective end coated with silver by Tollen's test. The reflective sensor was used to conduct five fixed liquid levels experiment for LPFG Nos. 1–5 and their corresponding liquid levels are 22, 42, 62, 82, and 102 cm. For each liquid level experiment, the sensing measurements were conducted five times for both measurements in air and immersed in water, respectively. The experimental findings showed that the reflective sensor was successfully to measure five liquid levels and it had at least 100-cm liquid level measurement capacity. The mechanism of the fluid-flow velocity sensor was based on analyzing the relationship among the optical power, time, and the LPFG's length. For the fluid-flow velocity measurement, there were two types of fluid-flow velocity measurements: inflow I and II (dry-to-wet process), as well as drainage (wet-to-dry process). Based on the results, the differences between the LPFG-based fluid-flow velocities (*v_LPFG_*) and the measured average fluid-flow velocities (*v_Measured_*) were found in the range of 8.7–12.6%. This reflective sensor is more suitable than conventional LPFG sensors (sensing with transmitted light) for the widespread use on structural sensing and monitoring applications in civil engineering. It is feasible that the reflective sensor has potential to measure liquid level and fluid-flow velocity without modifying LPFGs or coating chemical compounds.

We have successfully demonstrated the feasibility of a mobile LPFG liquid level sensor and a reflective LPFGs-in-series sensor possessing ability to successfully yield a comparable liquid level and fluid-flow velocity sensing performance. These two types of simple and low-cost fiber-optic sensor is expected to benefit the development and application of civil, hydraulic, and agronomy engineering, such as in liquid level and fluid-flow velocity monitoring for hydraulic structures and paddy fields, as well as in liquid level monitoring of tanks or reservoirs for industrial sectors.

## Figures and Tables

**Table 1.** Constants of upper and lower control limits for the *X*-bar charts, *s* charts, and *R* charts.

**Sample size**	***X-bar* chart constants**		***R* chart constants**		***s* chart constants**	
*n*	*A_2_*	*A_3_*	*D_3_*	*D_4_*	*B_3_*	*B_4_*
10	0.308	0.975	0.223	1.777	0.284	1.716
